# Diversity and distribution of sulfur metabolic genes in the human gut microbiome and their association with colorectal cancer

**DOI:** 10.1186/s40168-022-01242-x

**Published:** 2022-04-19

**Authors:** Patricia G. Wolf, Elise S. Cowley, Adam Breister, Sarah Matatov, Luke Lucio, Paige Polak, Jason M. Ridlon, H. Rex Gaskins, Karthik Anantharaman

**Affiliations:** 1 Institute for Health Research and Policy, University of Illinois at Chicago, Chicago, IL USA; 2 University of Illinois Cancer Center, University of Illinois at Chicago, Chicago, IL USA; 3 Department of Animal Sciences, University of Illinois Urbana-Champaign, Urbana, IL USA; 4 Division of Nutritional Sciences, University of Illinois Urbana-Champaign, Urbana, IL USA; 5 Department of Bacteriology, University of Wisconsin-Madison, Madison, WI USA; 6 Microbiology Doctoral Training Program, University of Wisconsin-Madison, Madison, WI USA; 7 Carl R. Woese Institute for Genomic Biology, University of Illinois Urbana-Champaign, Urbana, IL USA; 8 Cancer Center at Illinois, University of Illinois Urbana-Champaign, Urbana, IL USA; 9 Department of Biomedical and Translational Sciences, University of Illinois Urbana-Champaign, Urbana, IL USA; 10 Department of Pathobiology, University of Illinois Urbana-Champaign, Urbana, IL USA

**Keywords:** Colorectal cancer, Sulfur metabolism, Human microbiome, Gut, Cysteine, Taurine, Hydrogen sulfide, Metagenomics

## Abstract

**Background:**

Recent evidence implicates microbial sulfidogenesis as a potential trigger of colorectal cancer (CRC), highlighting the need for comprehensive knowledge of sulfur metabolism within the human gut. Microbial sulfidogenesis produces genotoxic hydrogen sulfide (H_2_S) in the human colon using inorganic (sulfate) and organic (taurine/cysteine/methionine) substrates; however, the majority of studies have focused on sulfate reduction using dissimilatory sulfite reductases (Dsr).

**Results:**

Here, we show that genes for microbial sulfur metabolism are more abundant and diverse than previously observed and are statistically associated with CRC. Using ~ 17,000 bacterial genomes from publicly available stool metagenomes, we studied the diversity of sulfur metabolic genes in 667 participants across different health statuses: healthy, adenoma, and carcinoma. Sulfidogenic genes were harbored by 142 bacterial genera and both organic and inorganic sulfidogenic genes were associated with carcinoma. Significantly, the anaerobic sulfite reductase *(asr)* genes were twice as abundant as *dsr*, demonstrating that Asr is likely a more important contributor to sulfate reduction in the human gut than Dsr. We identified twelve potential pathways for reductive taurine metabolism and discovered novel genera harboring these pathways. Finally, the prevalence of metabolic genes for organic sulfur indicates that these understudied substrates may be the most abundant source of microbially derived H_2_S.

**Conclusions:**

Our findings significantly expand knowledge of microbial sulfur metabolism in the human gut. We show that genes for microbial sulfur metabolism in the human gut are more prevalent than previously known, irrespective of health status (i.e., in both healthy and diseased states). Our results significantly increase the diversity of pathways and bacteria that are associated with microbial sulfur metabolism in the human gut. Overall, our results have implications for understanding the role of the human gut microbiome and its potential contributions to the pathogenesis of CRC.

Video abstract

**Supplementary Information:**

The online version contains supplementary material available at 10.1186/s40168-022-01242-x.

## Background

The human gut is a dynamic nutrient-rich environment that harbors a diverse metabolically active microbial community. Human health and disease are inextricably linked to microbial composition; however, much remains unknown regarding the functional capacity of human gut microbes [1–3]. This has manifested in bacteria or their niches being loosely characterized as “beneficial,” “commensal,” or “deleterious,” which is problematic as microbial functionality is often species-specific and microbes are capable of metabolic shifts based on available substrates [4, 5]. Genomic approaches have enabled the rapid discovery of novel bacteria whose functional characteristics have yet to be characterized [6]. These discoveries have filled gaps in knowledge regarding the metabolic capacity of human gut microbes and allow the design of hypothesis-driven interventions that create beneficial shifts in microbial communities. This approach may have particularly important implications in human diseases for which associations between microbial composition, dietary intake, and disease risks have been established.

For example, there is strong evidence linking a diet high in red and processed meat with colorectal cancer (CRC) [7]. In addition, bacteria capable of producing hydrogen sulfide (H_2_S) are associated with a western diet [8, 9], colonic inflammation [10], and CRC [11–19]. At micromolar concentrations [20], endogenously produced H_2_S can act as a vasorelaxant [21], reduce endoplasmic reticulum stress [22], and prevent apoptosis [23]. At millimolar concentrations, as commonly found in the colon, H_2_S inhibits cytochrome oxidase causing reductive stress and is genotoxic [24–27]. Previous works investigating microbial sulfidogenesis in the human gut have mostly focused on sulfate-reducing bacteria (SRB) that perform inorganic sulfur metabolism [28]. However, recent evidence indicates that organic sulfur metabolism by gut bacteria may be a key mechanism linking diet and CRC [29]. Indeed, CRC-associated bacteria have been shown to produce H_2_S via metabolism of sulfur amino acids [6], and the taurine metabolizing *Bilophila wadsworthia* was found previously to be a significant indicator of CRC [11]. Consumption of a diet high in red and processed meat increases colonic concentrations of organic sulfur, which may increase colonic concentrations of microbially derived H_2_S to genotoxic levels [11, 30, 31]. This suggests that sulfur metabolism in the human gut microbiome may be more widespread than originally believed and exposes current gaps in our knowledge of the metabolic functions of CRC-associated bacteria.

Thus, the objective of this study was to use genomic and metagenomic tools to gain a greater understanding of the sulfidogenic capacity of the human gut microbiome. To do so, we investigated the prevalence of sulfidogenic genes in gastrointestinal bacterial genomes, established a network of sulfur metabolic transformations, and identified novel sulfidogenic bacteria. Using newly developed gene databases, 16,936 publicly available bacterial metagenome-assembled genomes (MAGs) from human gut microbiomes were mined to compare the relative contribution of inorganic and organic sulfidogenic genes to microbial sulfur metabolism. Gene presence was then compared among disease states in five CRC microbiome studies to evaluate potential contributions of microbial H_2_S production to CRC risk. This study provides the most comprehensive analysis of microbial sulfur metabolism in the human gut to date and thereby provides a platform for hypothesis-driven experiments characterizing the role of sulfur metabolites in CRC and other inflammatory-associated gut disorders.

## Results

### Common pathways of microbial sulfur metabolism are prevalent in human gut microbiomes

**Fig. 1:**
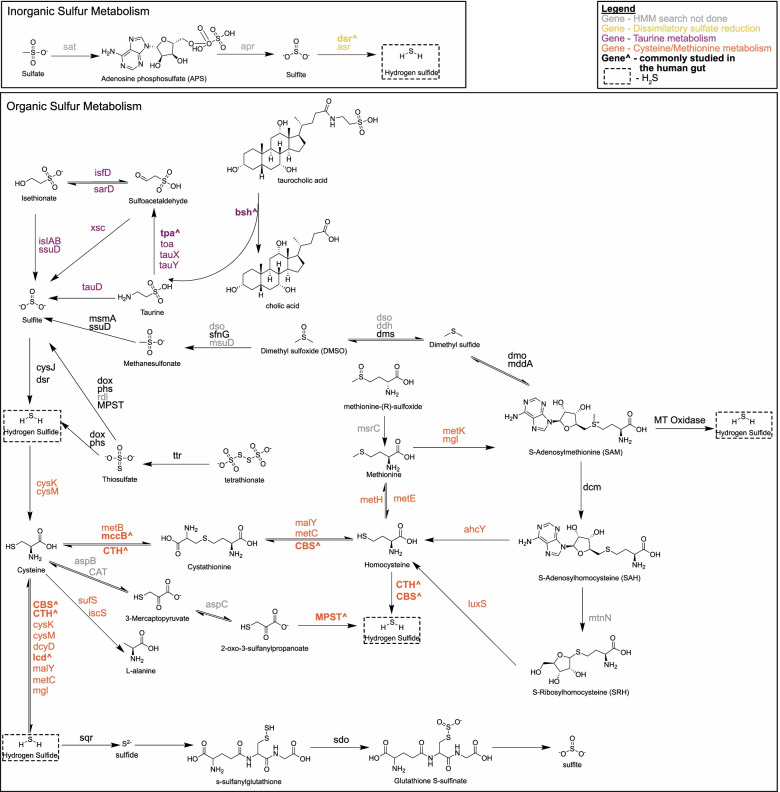
Potential microbial sulfur transformations in the human gut microbiome. Microbial sulfur metabolism results in the production of genotoxic H_2_S (dashed box) via metabolism of inorganic sulfate (yellow) or organic sulfur amino acids like cysteine and methionine (maroon), or taurine (orange). Previous studies of microbial sulfidogenesis in the human gut have focused mainly on genes harbored by *Bilophila*, *Fusobacterium*, and the sulfate-reducing bacteria (bolded with a “caret”). All genes listed were analyzed in this study except those listed in gray. Reactions are not balanced and only the main sulfur component reactants and products are shown. Some intermediate steps are not shown

To understand the diversity, distribution, and ecology of microbial sulfur metabolism in the human gut and its implications in disease, we investigated the complex pathways for sulfur transformations (Fig. 1). Stool shotgun metagenomic sequence data were used from 5 publicly available studies that investigated the gut microbiome in healthy subjects and patients who had adenoma or carcinoma of the colon. Collectively, 265 healthy participants, 112 participants with adenoma, and 290 participants with carcinoma were examined. Participant location, associated metadata, and study references are listed in Table 1 [17, 32–35]. A total of 16,936 bacterial metagenome-assembled genomes (MAGs) were recovered from a previous study that used standardized bioinformatic pipelines for metagenome assembly [36]. A concatenated ribosomal protein tree was created to examine the full diversity of the samples used in this study with the majority of genomes being classified in the phyla Firmicutes, Proteobacteria, and Actinobacteria (Fig. S1). Open reading frames (ORFs) were then predicted followed by homology-based identification of 79 genes associated with microbial sulfur metabolism (Table S1) using available and custom Hidden Markov Models (HMMs). These analyses revealed the breadth of the microbial sulfur metabolic potential in the human gut and its associations with CRC (Table S2, Fig. S1).

**Table 1:** Overview of original datasets used for this study

Study	Country of participant recruitment	Disease state	Number of participants	Number of MAGs
Feng [32]	Austria	Control/healthy	61	1690
		Adenoma	47	1421
		Carcinoma	46	1418
Hannigan [34]	USA, Canada	Control/healthy	26	101
		Adenoma	23	57
		Carcinoma	26	78
Vogtmann [33]	USA	Control/healthy	58	1863
		Adenoma	0	0
		Carcinoma	52	1622
Yu [35]	China	Control/healthy	54	1397
		Adenoma	0	0
		Carcinoma	75	1910
Zeller [17]	France	Control/healthy	66	1839
		Adenoma	42	956
		Carcinoma	91	2584
Totals		Control/healthy	265	6890
		Adenoma	112	2434
		Carcinoma	290	7612

To understand the prevalence of microbial sulfur metabolism in a representative human gut microbiome, 514 fecal microbial genomes were obtained from the Human Microbiome Project (HMP) and surveyed for common metabolic pathways associated with H_2_S production from cysteine, taurine, and sulfate/sulfite. Functional genes encoding proteins for cysteine metabolism made up the majority of sequences and included cystathionine-β-lyase (*malY*, *metC*) (11.8%), cystathionine-β-synthase (*CBS*) (27.5%), cysteine desulfhydrase (*lcd*) (2.6%), d-cysteine desulfhydrase (*dcyD*) (17.9%), and methionine-γ-lyase (*mgl*) (14.7%). In contrast, dissimilatory sulfite reductases (*dsrAB*) catalyzing the final step of sulfate and taurine respiration to form H_2_S, made up only 18.2% of identified genes (Fig. S2). In total, 313 sulfidogenic genes were identified from 183 bacterial genomes (35.6% of total genomes), and spanned across six phyla including Proteobacteria (36.1%), Firmicutes (26.2%), Bacteroidetes (20.2%), Fusobacteria (15.8%), Actinobacteria (1.1%), and Synergistetes (0.5%). This key finding demonstrates that pathways for sulfur metabolism were prevalent in human gut microbiome genomes and that cysteine may be an underestimated substrate for microbial sulfur metabolism in the gut.

### Genes for anaerobic sulfite reductases (asrABC) are more prevalent than dissimilatory sulfite reductases (dsrAB) in the human gut

Of the limited literature regarding human colonic sulfidogenic bacteria, the most well studied are the SRB which are capable of reducing inorganic sulfate supplied exogenously by diet or endogenously by degradation of sulfated bile acids and mucins (estimated 1.5–16 and 0.96–2.6 mmol/day respectively) [28, 37–40]. Two enzymes are able to complete the final step of the reaction which catalyzes the six-electron reduction of sulfite to H_2_S — dissimilatory sulfite reductase (Dsr) and anaerobic sulfite reductase (Asr) (Fig. 1). It has been proposed that sulfite reduction takes place as a series of two electron transfers to DsrAB from the DsrMKJOP complex via DsrC [41]. Genes for the Dsr pathway, *dsrAB*, are highly conserved among SRB and diversely distributed among phyla in environmental samples [42]. However, culture and PCR-based studies of SRB diversity in human stool and colonic mucosa indicate that *dsrAB* is harbored by only five resident genera namely *Bilophila* spp., *Desulfovibrio* spp., *Desulfobulbus* spp., *Desulfobacter* spp., and *Desulfotomaculum* spp. [43–46].

**Fig. 2:**
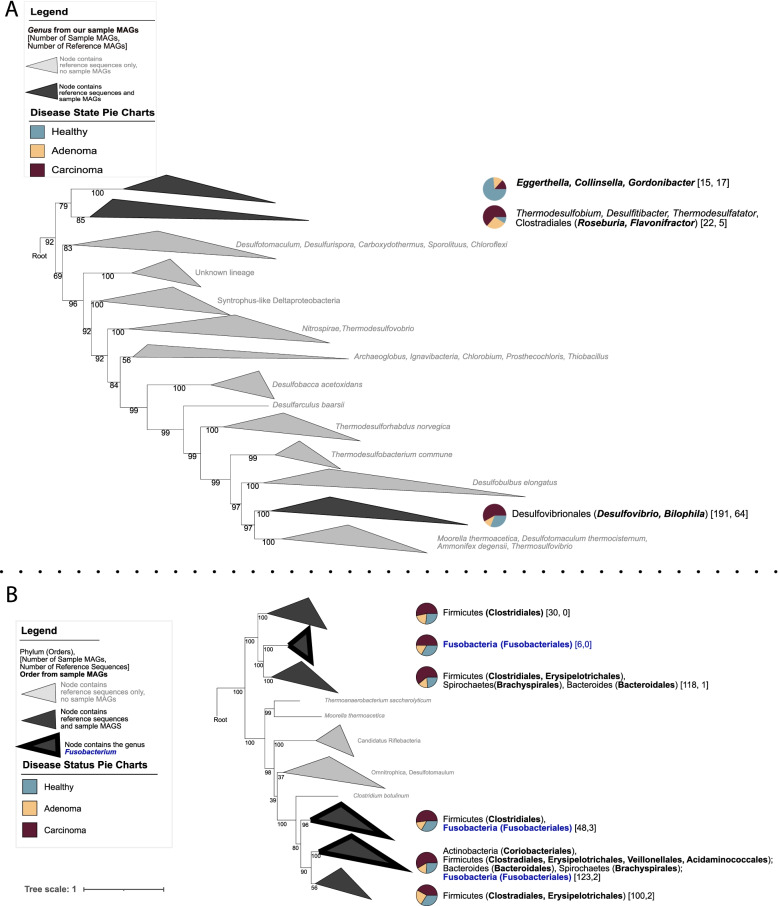
Concatenated protein trees for dissimilatory sulfate reduction pathways. **A** Concatenated protein tree showing the diversity of bacteria that possess genes for the final enzyme of the dissimilatory sulfate reduction pathway — dsrAB. **B** Concatenated protein tree showing the diversity of bacteria that possess genes for anaerobic sulfite reductase (asrABC), an enzyme also capable of dissimilatory sulfate reduction. Gray clades only contain reference sequences, darker gray clades contain reference sequences and sequences from this study. Bracketed numbers indicate the sequence origin within each clade: (number of sequences from our study, number of sequences from references). Bacterial genera (dsr) or orders (asr) originating from study samples are bolded. Pie charts indicate the disease state associated with sequences within each clade with blue indicating healthy, yellow adenoma, and maroon carcinoma. Clades outlined in black contain *Fusobacterium* sequences

Within our database, *dsrAB* genes were present in 121 MAGs of 16,936 bacterial MAGs (<1% of total MAGs), from human gut samples and in 17.4% of the total subjects (Table S3). Taxonomic classification demonstrated genera commonly associated with Dsr activity in the gut microbiota were represented including *Desulfovibrio* spp. and *Bilophila* spp. In addition, six genera were revealed that are not commonly ascribed as human gut SRBs namely *Collinsella* spp., *Eggerthella* spp., *Enterococcus* spp., *Flavinofracter* spp., *Gordonibacter* spp., and *Roseburia* spp. (Tables S2 and S4). Since previous phylogenetic analyses in environmental samples indicated that *dsrAB* acquisition was often the result of multiple lateral gene transfer events [42], a concatenated gene tree was generated with a reference database of *dsrAB* sequences to observe the consensus phylogeny of *dsrAB* sequences in human gut bacteria. *DsrAB* sequences from sample MAGs separated into three distinct clusters which corresponded with the respective phyla: Actinobacteria, Firmicutes, and Proteobacteria (Fig. 2A). This suggests that the lateral gene transfer of *dsrAB* may be less common in SRBs of the human gut than those observed in environmental studies [42].

While SRBs may be the most well-studied sulfur-metabolizing bacteria of the human gut microbiota, studies have focused mainly on bacteria harboring Dsr enzymes. Similar to Dsr enzymes, Asr performs a 6-electron reduction of sulfite to H_2_S (Fig. 1). A recent analysis of environmental diversity of the *asrABC* complex revealed its presence in common residents of the gut microbiome including *Fusobacterium nucleatum* and *Clostridium intestinale* [47]. To determine if gut bacteria harbor *asrABC*, we searched the MAG database, revealing that *asrABC* genes were more prevalent in all MAGs and participants than *dsrAB* genes. The *asrABC* genes were present in approximately 2% of the total MAGs (388, 390, and 375 MAGs, respectively), and approximately 35% of the total subjects (Table S3). Intriguingly, less than a quarter of the subjects that harbored *asrABC* genes also possessed genes for *dsrAB* (53 of 239 subjects). Taxonomic assignments showed thirty-one genera possessed *asrABC*, spread among five phyla including Actinobacteria, Firmicutes, Fusobacteria, Spirochaetes, and a phylum not previously shown to possess *asrABC* — Bacteroidetes [47]. A consensus tree of reference and sample MAG concatenated *asrABC* sequences revealed clustering of *asrABC* genes across six nodes that were distinct from sample MAG phylogeny. Notably, *asrABC* genes possessed by *Fusobacterium* spp. did not cluster together, but were observed in three separate nodes suggesting this pathway was acquired via multiple lateral gene transfers (Fig. 2B). Together, these data indicate that Asr enzymes may be more important contributors to sulfate and sulfite reduction than Dsr in the human gut and that bacteria acquired these enzymes via a divergent phylogenetic history.

### Diverse bacteria harbor pathways for taurine metabolism

Sulfonates are organic sulfur compounds with a SO_3_^−^ moiety that are abundant in marine sediments and detergents and play an important role in the environmental sulfur cycle [48]. While less is known about the role of sulfonates in the human intestine, there is evidence that the metabolism of sulfonates like isethionate and taurine is performed by resident gut microbes [49, 50]. Microbial taurine metabolism has gained considerable interest after it was implicated as a potential dietary mechanism of colitis and CRC disparities [10, 11]. Representing 18.3% of the total free amino acids in the colonic mucosa (13.6 ± 0.5 mmol/kg), taurine is the second most abundant free amino acid in this tissue [51]. Taurine is provided as a substrate to the human gut microbiota either directly through diet or through hydrolysis of taurine-conjugated bile acids by the enzyme bile salt hydrolase (BSH). Excess consumption of taurine and cysteine increases tauro-conjugation of secreted bile acids, thus potentially providing additional substrates for bacteria with BSH activity [29]. The *bsh* gene was identified to be present in 15% of total MAGs, and in 89% of the total subjects (Table S3). This prevalence was unsurprising, as it has been proposed that hydrolysis of conjugated bile acids may be a detoxification strategy to decrease bile acid toxicity [52], or may serve as a source of nutrients for microbial growth and energy metabolism [28].

Once liberated via BSH or made available through dietary intake, taurine can be metabolized by gut bacteria via oxidative or reductive pathways. Taurine oxidation using the enzyme taurine dioxygenase (TauD) is generally not considered as a source of H_2_S production in the anaerobic environment of the human colon. However, previous studies have reported an increase of aerotolerant bacteria adherent to the mucosal surface suggesting a luminal gradient of oxygen provided by host tissues [53]. Assessment of *tauD* gene abundance demonstrated that while the enzyme was present in only 1% of the total MAGs, these MAGs were present in 23% of subjects (Table S3). As expected, *tauD* genes were present in six facultative genera (*Escherichia* spp., *Enterobacter* spp., *Citrobacter* spp., *Morganella* spp., *Hafnia* spp., and *Raoultella* spp.); however, none of these genera possessed genes for anaerobic or dissimilatory sulfite reduction (Table S4). Thus, TauD appears to be primarily used for taurine assimilation and not H_2_S production.

**Fig. 3:**
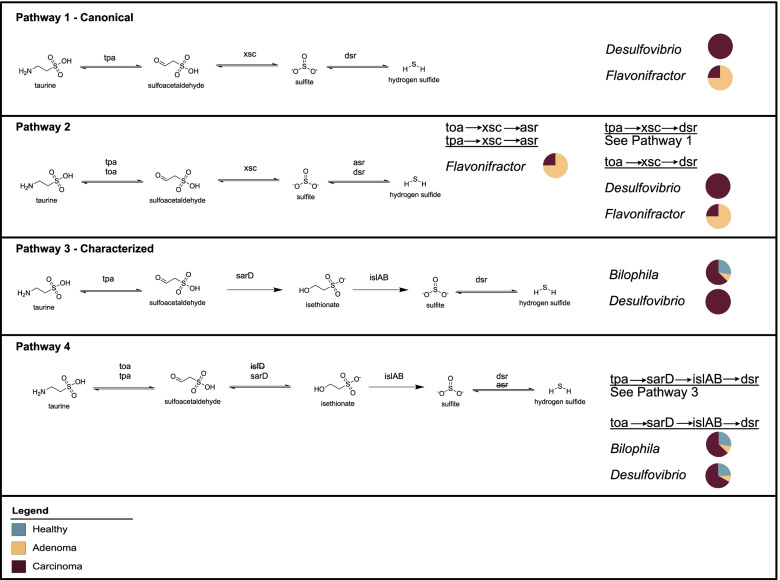
Characterized and proposed pathways of microbial taurine reduction to H_2_S. Pathway 1 — the canonical pathway of taurine reduction in *Bilophila wadsworthia*. Pathway 2 — putative 3-step reactions for taurine reduction analyzed in this study. Pathway 3 — the recently characterized pathway for taurine reduction in *Bilophila wadsworthia*. Pathway 4 — putative 4-step reactions for taurine reduction analyzed in this study. No complete pathways were found involving genes that are struck through. Genera possessing genes for each complete pathway are listed. Only genera listed were found to have complete pathways. Pie charts indicate the disease state associated with MAGs of each genus with the specified pathway with blue indicating healthy, yellow indicating adenoma, and maroon indicating carcinoma

The only known bacterium to possess the reductive pathway of taurine metabolism in the human gut is *B. wadsworthia*. However, given the taurine-rich environment of the colon, it is likely that other bacteria capable of performing this metabolism remain to be discovered. Thus, to identify candidates that may have the capacity to produce H_2_S from taurine in an anaerobic environment, HMM searches of the described cohorts were performed targeting pathways as shown in Fig. 3. Pathway 1 describes the putative taurine reduction pathway previously thought to be possessed by *B. wadsworthia* [28] Our search confirmed that this pathway was not possessed by *Bilophila* spp., as recently described [54]. Instead, analyses revealed two genera that harbor genes for this pathway, namely *Desulfovibrio* spp. and *Flavonifractor* spp. The first step of taurine reduction involves the liberation of the nitrogenous group from taurine producing pyruvate or 2-oxoglutarate via the enzymes taurine-pyruvate aminotransferase (Tpa) or taurine-2-oxoglutarate transaminase (Toa), respectively (pathway 2). For all MAGs that possess the latter two genes of this pathway, the genes *tpa* and *toa* co-occur. Notably, *Flavonifractor* spp. also harbor genes for the AsrABC complex, indicating an alternative final step of the taurine reductive pathway (pathway 2). Additionally, evaluation of three-step pathway combinations revealed 34 genera that possessed the first and final pathway steps, suggesting the pervasiveness of metabolic cooperation in the gut and potentially revealing targets for the identification of novel sulfoacetaldehyde acetyltransferases (Xsc) (Fig. 3) (Tables S4 and S5).

Pathway 3 represents the recently characterized pathway for taurine reduction in *B. wadsworthia* (Fig. 3) [54]. Gene searches corroborated that *Bilophila* spp. harbored all four genes in this newly defined pathway. However, contrary to what was recently reported, all four genes were also observed in *Desulfovibrio* spp. Since MAGs are unable to allow for granularity at the species level, future work is needed to determine if this pathway is indeed more widespread in resident *Desulfovibrio* species of the human gut. Seven MAGs annotated as *Desulfovibrio* spp. and *Bilophila* spp. also possessed *toa* in the absence of *tpa*; together, this indicates an alternative first step to this pathway (pathway 4). Notably, unlike the three-step pathway 2, MAGs that possess all genes for the four-step pathway harbor only *dsrAB* and not *asrABC*. In addition, evaluation of four-step pathway combinations revealed seven genera missing either the second or third step of the pathway revealing additional targets for gene discovery (Fig. 3) (Tables S4 and S5).

### Cysteine and methionine are understudied and abundant sources of microbially derived H_2_S in the human gut

Cysteine is a conditionally essential amino acid, which is provided to the intestine directly by diet or the decomposition of methionine (Fig. 1). Methionine restriction alters the microbial composition of the gut and downregulates inflammatory pathways related to oxidative stress [55, 56]. In addition, the production of H_2_S via cysteine degradation supports microbial growth and protects from oxidative stress in response to antibiotic treatment [57]. Cysteine-metabolizing bacteria are implicated as a source of oral abscess, breath malodor, and delayed wound healing in the oral cavity [58–61], and have been repeatedly associated with CRC [12, 14, 16, 17]. Of late, *F. nucleatum* has been of particular interest in CRC [14, 16, 62]. Studies have demonstrated the association between *F. nucleatum* and the tumor surface in a subset of CRC [12, 14, 63], *F. nucleatum* DNA in CRC tumors correlate with reduced survival [64, 65], and two subspecies of *F. nucleatum* (*vincentii* and *animalis*) have been proposed as part of a microbial signature for fecal-based CRC classification [17]. However, few studies appreciate that *F. nucleatum* is sulfidogenic, and many other bacteria can also produce H_2_S from cysteine in the human gut.

To gain an appreciation of the abundance of cysteine metabolism within the human microbiome, MAGs were searched for the following genes associated with cysteine metabolism *dcyD*, *malY*, *metC*, and *mgl*, as well as cysteine synthase (*cysK*, *cysM*), cysteine desulfurase *(iscS*, *sufS*), and cystathionine-γ-synthase (*metB*), and the three human orthologs cystathionine-β-synthase (*CBS*), cystathionine-γ-lyase (*CSE/mccB*), and 3-mercaptopyruvate sulfurtransferase (*3MST*). Genes for upstream pathways of methionine and homocysteine metabolism were also analyzed (Fig. 1, Table S1). Searches revealed that all cysteine-metabolizing genes were highly present, with *cysK*, *lcd*, *malY*, and *sufS* observed at least once in over 96% of subjects (Table S3). Even those genes that were observed in only 2–5% of the total MAGs — namely *dcyD*, *metC*, *cysM*, and *mgl* — were still observed to be present in at least 40% of subjects (Table S3). In accordance with this, genes for microbial pathways for methionine metabolism were also highly present in subjects, indicating that methionine may be an important source of microbial-derived cysteine in the human gut (Table S3). In addition to being abundantly present, genes for cysteine metabolism were also diversely distributed among 13 phyla and 141 genera. Among these, 84 genera have not been previously characterized as being sulfidogenic (Table S4). This may be particularly important in the context of a western diet, as studies using fecal homogenates demonstrate higher production of H_2_S from organic sulfur amino acids compared to inorganic sulfate [66], and higher protein intake increases the ileal output of protein (2.69 vs. 7.45 g/day) and free amino acids (6.90 vs. 20.48 μmol/mL) [67]. Although additional work is needed to comprehensively resolve cysteine metabolism, together these data indicate that the sulfur amino acids cysteine and methionine may be an understudied and abundant source of microbially derived H_2_S in the human gut.

### Microbial sulfur metabolism is statistically associated with colorectal cancer

**Fig. 4:**
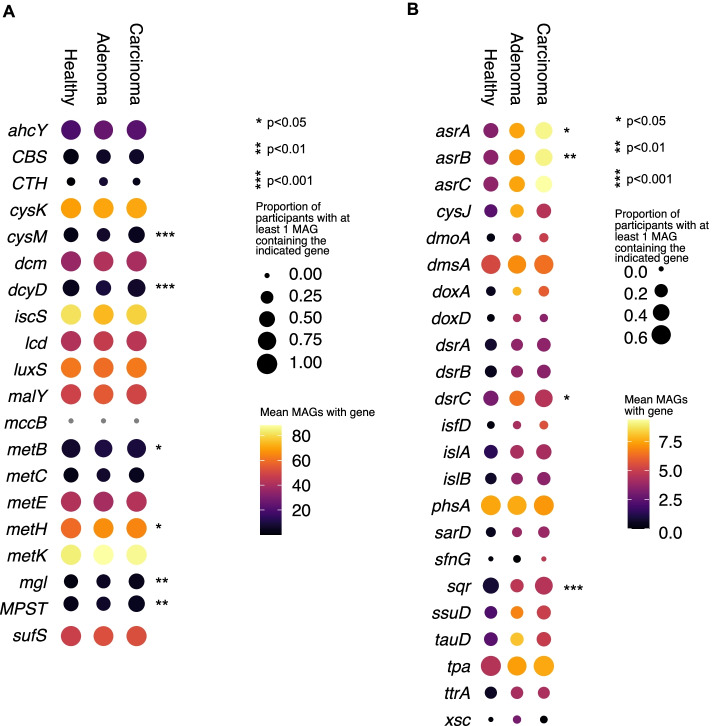
Genes for microbial sulfur metabolism are abundant and significantly associated with colorectal cancer. Dot plots of selected genes related to microbial cysteine and methionine metabolism (**A**) and taurine and sulfur metabolism (**B**) across three disease states: healthy, adenoma, and carcinoma. The size of each dot indicates the proportion of participants in each disease state with at least one copy of the indicated gene in their bacterial MAGs and the color of each dot indicates the mean number of MAGs with that gene in the subset of participants that have at least one copy of the gene. Genes that have a non-random distribution across disease status as analyzed by chi-squared analysis are indicated by asterisks. *p*-value corrections were done using the Benjamini-Hochberg (BH) Procedure

Sulfate-reducing bacteria and H_2_S have been implicated in CRC pathogenesis [24, 25, 30, 68, 69]; however, associations of other microbial sulfur metabolism genes with CRC have been less well studied. The proportion of participants in each health status (healthy, adenoma, or carcinoma as evaluated by colonoscopy) category with at least one copy of each gene was determined along with the proportion of the total number of MAGs in each health status with the gene (Fig. 4, Table S3). Genes involved with cysteine and methionine metabolism were generally abundant regardless of health status (Fig. 4A, size of dots) and abundant in many MAGs (Fig. 4A, color of dots). Genes involved in sulfur and taurine metabolism were variable in their distribution among participants in the three health states and in MAGs (Fig. 4).

To determine if sulfur metabolism was associated with CRC, we conducted statistical tests to study the distribution of sulfur genes in participants across each health status. Genes for tetrathionate metabolism, which was previously implicated as an electron acceptor provided by gut inflammation [70], were present in less than 1% of MAGs and were not significantly different among the three health statuses. Genes involved in cysteine and methionine metabolism including *cysM*, *dcyD*, *mgl*, *metB*, *metH*, and *sdo* exhibited distributions that were statistically significantly different (at least a *p*-value < 0.05) among participants in the three health states (Fig. 4A, Table S6, Figs. S3 and S4), with *sdo*, *cysM*, *mgl*, *metB*, and *metH* being more likely to be found in carcinoma. While 92 genera with cysteine-metabolizing genes have been associated with CRC previously, our results indicate that this association may involve sulfidogenesis. Indeed, MAGs for cysteine-metabolizing genes were pervasive in genera most commonly associated with CRC, corroborating recent work that observed that genes for cysteine metabolism were significantly more abundant in subjects with CRC [71] (Fig. S5). Sulfur and taurine metabolism genes, *asrA*, *asrB*, *dsrC*, and *sqr*, had distributions that were statistically significantly different (at least a *p*-value < 0.05) among the three health states with all being more likely to be found in carcinoma (Fig. 4B, Table S6, Figs. S3 and S4).

Intriguingly, for genes related to dissimilatory sulfate reduction, different organisms exhibited different clustering patterns based on both phylogeny and disease state. For the Dsr pathway, the majority of *Desulfovibrio* spp., *Bilophila* spp., *Flavinofracter* spp., and *Roseburia* spp. were harbored by participants with carcinoma while the majority of *Collinsella* spp., *Eggerthella* spp., and *Gordonibacter* spp. originated from healthy participants (Fig. 2A — pie charts). For the Asr pathway, the majority of sequences were recovered from participants with carcinoma (Fig. 2B — pie charts). These data along with the associations presented in Fig. 4B demonstrate that genes for sulfate reduction are associated with carcinoma.

To gain a deeper understanding of the ecology of microbial sulfur metabolism during colorectal carcinogenesis, we first determined the associations of sulfidogenic genes among different CRC stages from the three datasets that reported staging data and then investigated growth rates of selected taxa previously considered to be microbial markers of CRC [11, 17]. The likelihood of patients having sulfidogenic genes was not significantly different among stages (Supplemental Table 7). However, participants were more likely to have genes for inorganic sulfur metabolism in earlier stages of CRC, while participants having genes for organic sulfur metabolism was uniformly high independent of the stage (Fig S6). *Mgl* — an organic sulfur-metabolizing gene — was increasingly present across advancing cancer stages. This is intriguing as *mgl* has been shown recently to be the most highly transcribed of cysteine-metabolizing genes [71]. Growth rate analysis of bacterial indicator species of CRC did not reveal statistically significant differences among disease states for any species analyzed; however, this analysis was restricted by the limited presence of these species within healthy subjects (Fig. S7, Tables S8 and S9). Future analyses of microbial species that are present across all disease states are needed to determine if other keystone species play a role in CRC pathogenesis than determined previously by taxa abundance.

## Discussion

There is compelling evidence implicating microbial H_2_S production as an environmental trigger of CRC; however, ongoing studies investigating this link have been hampered by the field’s incomplete knowledge of sulfur metabolism within the human gut. Here, we performed a comprehensive characterization of the functional capacity of the human gut microbiome to conduct sulfur transformations and produce H_2_S. Our investigation into the diversity and ecology of inorganic sulfur metabolism pathways observed that highly conserved functional genes encoding the final step of the sulfate reduction pathway — *dsrAB* — was harbored by six genera not typically targeted as SRB. Additionally, this investigation revealed that *asrABC*, which encodes an enzyme with the same biochemical activity to Dsr, was both twice as abundant in total MAGs and was present in twice as many subjects as *dsrAB*. Together, these data highlight that genes for inorganic sulfur metabolism in the human gut are more widespread than previously established and that *asrABC* may be an important marker to measure the capacity of microbial sulfate reduction within cohorts. Since the diversity of microbial sulfatases has not been characterized, studies that compare substrate specificity and the catalytic efficiencies of these enzymes are needed to truly understand the implications of this expanded view of inorganic sulfur metabolism in the human gut.

While previous investigations of microbial H_2_S production and human disease have focused on SRB, bacteria that metabolize sulfur from organic sources have been consistently associated with CRC risk. Prior to this work, *Bilophila wadsworthia* was the only bacterium known to produce H_2_S via taurine respiration. However, our analysis revealed *Desulfovibrio* spp. harbors genes for the characterized 4-step reductive pathway of taurine respiration, and an exploration of twelve taurine reduction pathways revealed two genera with genes for the complete 3-step reduction pathway. Further, 41 unique genera were found to have nearly complete 3- and 4-step pathways, disclosing microbial targets for novel enzyme discovery or investigations of cooperative taurine metabolism. Finally, analyses of diverse pathways for microbial cysteine and methionine degradation revealed these sulfidogenic genes were distributed among diverse bacterial phyla and were abundantly present among subjects. Collectively, these analyses demonstrate that bacteria harboring pathways for organic sulfur metabolism are pervasive in the human gut and likely constitute the most abundant source of microbially derived H_2_S.

**Fig. 5:**
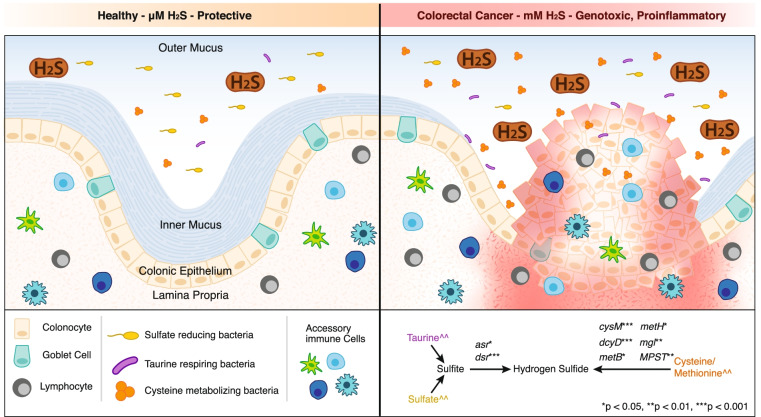
Organic sulfur metabolism by gut bacteria may be a key mechanism linking a western diet and CRC risk. The degradation of sulfomucins by mucolytic bacteria is a key source of inorganic sulfate for sulfate-reducing bacteria. At micromolar concentrations, basal production of H_2_S through inorganic sulfate reduction exerts beneficial effects including gut barrier protection and fermentative hydrogen disposal. Intake of a western diet abundant in red and processed meat amplifies the production of taurine conjugated bile acids and increases colonic exposure to dietary sulfur amino acids (taurine, methionine, cysteine). In the context of a western diet, the metabolism of organic sulfur amino acids by gut microbes drives the production of H_2_S to genotoxic and pro-inflammatory levels (mM concentration). Simplified pathways demonstrate genes for sulfur metabolism that were significantly associated with CRC. The symbol '^' indicates inorganic sulfur sources primarily provided by sulfated bile acids and sulfamucins. The '^^' symbol indicates organic sulfur sources provided by dietary sulfur amino acids and conjugated bile acids

To determine whether sulfur metabolism was differentially associated along the colorectal carcinoma sequence (healthy → adenoma → carcinoma), the presence of sulfidogenic genes was compared between healthy subjects and patients with adenoma or carcinoma. Genes for both inorganic and organic sulfur metabolism were significantly associated with carcinoma, which is intriguing as both sulfate and taurine metabolism share a final metabolic step. These associations along with the pervasiveness of genes for organic sulfur metabolism in studied MAGs support the unique hypothesis that organic sulfur metabolism by gut bacteria is a key mechanism linking a western diet and CRC risk (Fig. 5). A limitation of our study is that our analyses utilized MAGs which do not use all available metagenomic reads as a consequence of assembly and binning. Thus, it is possible that the abundance of sulfur genes present in the human gut is even greater than reported herein. However, the use of MAGs enabled taxonomic information, reconstruction of full pathways, and calculation of growth rates for selected CRC associated bacteria, thus providing robust new information regarding microbial sulfur metabolism in the human gut. In addition, this work focused on the functional capacity of the gut microbiome for sulfur metabolism based on genetic content. Future work that demonstrates the metabolic activity of microbes and their enzymes in vitro, as well as the metabolic flux in a complex microbial community, is needed to verify these conclusions.

## Conclusions

Overall, the data demonstrate that genes for microbial sulfur metabolism are more diverse than previously recognized, are widely distributed in the human gut microbiome, and are significantly associated with CRC. These data expand what was previously known regarding the diversity of bacteria with the genetic capacity to perform sulfur metabolism, and indicate that genes for organic sulfur metabolism may be the most important contributor of H_2_S in the human gut. Our findings provide a foundation for future work characterizing the activity of sulfidogenic enzymes in diverse microbial species, exploring the expression of these genes as affected by health status, and examining microbial H_2_S induced tumorigenesis in animal models of CRC and human disease.

## Methods

### Genomic survey of sulfidogenic genes in Human Microbiome Project genomes

An initial genomic survey was performed using 514 gastrointestinal genomes obtained from the Human Microbiome Project (HMP) in Fall 2018 [72, 73]. Reference sequences were obtained from the National Center for Biotechnology Information (NCBI) using searches for sulfidogenic genes from known residents of the human gut including “cysteine desulfhydrase”, “Cdl”, “Lcd”, “cystathionine-beta-synthase”, “l-methionine–gamma-lyase”, “dissimilatory sulfite reductase”, “dsrA”, “dsrB”, and “dsrAB”. Searches of the HMP genes were then performed using BLAST (BLASTv2.8.1+) [74], and alignments with greater than 60% identity and a minimum query coverage of 40 amino acids were retained. To filter non-homologous proteins, gene hits were compared to KEGG.

### Sulfur pathway visualization

Sulfur cycle reactions were created in ChemDraw Prime 16.0 and further modified in Affinity Designer.

### Downloading MAGs and accessing metadata

The previously reconstructed MAGs from the five cohorts were downloaded from https://opendata.lifebit.ai/table/sgb.The associated file that was downloaded, “download_files.sh” was used to download all 16,936 genomes from the 5 studies [36]. The link to download the metadata (https://www.dropbox.com/s/ht0uyvzzal6exs2/Nine_CRC_cohorts_taxon_profiles.tsv?dl=0) was found at http://segatalab.cibio.unitn.it/data/Thomas_et_al.html from two studies [75, 76] and filtered to only include the 5 cohorts used in our study. For samples that had AJCC TNM (Tumor, Node, Metastasis) classification without a stage, the American Cancer Society guidelines to annotate stage based on TNM classification was used. For this study, all “high” (> 90% complete, < 5% contamination, < 0.5% strain heterogeneity) and “medium” (> 50% complete, < 5% contamination) quality MAGs were included [36, 77].

### Gene annotation of MAGs

Prodigal module (prodigal version 2.6.3) of METABOLIC was used to run multiple threads with the -p meta option to annotate open reading frames (ORFs) on all MAGs [78, 79].

### Sulfur gene identification in MAG database

HMM — Hidden Markov Model (HMM) searches for protein sequences were performed using either hmm profiles from KEGG or custom profiles against a concatenated file of predicted ORFs from all 16,936 MAGs used for this study with HMMSearch version 3.3.0 [80], using trusted cut-offs for all sulfur-related sequences (Table S1). All code and HMMs used in this study are available at https://github.com/escowley/HumanGutBacterialSulfurCycle.

### Microbial sulfur pathway literature search

To determine the breadth of current knowledge regarding characterized H_2_S production in human gut bacteria and their association with CRC, a literature search was performed on genera revealed in our analysis. For MAGs that possessed sulfidogenic genes, the annotations for the “closest genus” were recorded. Web searches were performed for each genus using the keywords “colorectal cancer,” “sulfide,” and “H_2_S.” Genera previously associated with CRC or who have species characterized previously to produce H_2_S are noted in Supplemental Table 4.

### Taxonomic classifications of MAGs

Taxonomic classifications, “closest” designators, were determined previously by binning the MAGs with a reference set of genomes [36]. To verify taxonomic classifications for MAGs, a concatenated ribosomal protein tree, GTDB-Tk classification, and 16S rRNA alignment were done. Taxonomic classifications from the original study, GTDB-tk, and 16S rRNA genes can be found in Supplemental Table 10. 16S rRNA sequences were extracted from the MAGs using the ssu_finder function of checkM (version 1.0.11) [81]. From the 16,936 MAGs, 2531 16S rRNA sequences were extracted. Extracted 16S rRNA sequences were classified using with SINA aligner (version v1.2.11) (general options — bases remaining unaligned at the end should be attached to the last aligned base, reject sequences below 70% identity) using the search and classify feature (search and classify options — min identity with query sequence 0.95, number of neighbors per query sequence 10, sequence collection used — Ref-NR, search kmer candidates 1000, lca-quorum 0.8, search k-mer len 10, search kmer mm 0, search no fast, taxonomies used for classification SILVA, RDP, GTDB, LTP, EMBL-EBI/ENA), which classifies 16S rRNA sequences based on the least common ancestor and the SILVA reference database (release 138.1) [82–84]. Taxonomy was assigned using GTDB-Tk (version 1.3.0) with database release 95 with the classify_wf function [85–87].

For the concatenated ribosomal protein tree, a curated Hidden Markov Models (HMM) database for single-copy ribosomal proteins (rpL2, rpL3, rpL4, rpL5, rpL6, rpL14, rpL14, rpL15, rpL16, rpL18, rpL22, rpL24, rpS3, rpS3, rpS8, rpS10, rpS17, rpS19) [88] was used to identify these genes in all MAG using hmmsearch (version 3.3.0) using noise cutoffs (--cut_nc) [80]. Once identified, the protein sequences were extracted from the predicted ORFs and imported into Geneious Prime (v 11.1.5). Each sequence was aligned with a reference set using MAFFT (v 7.450, parameters Algorithm: Automatic, Scoring Matrix: BLOSUM62, Gap penalty: 1.53, Offset value: 0.123) [89]. The alignments were manually trimmed and a 95% gap masking threshold was applied to the resulting alignment. The resulting alignments were concatenated for each gene set. The concatenated alignments were exported in fasta format from Geneious and used as the input for making phylogenetic trees with IQTree using version 1.6.9 (-nt AUTO -m MFP -bb 1000 -redo -mset WAG,LG,JTT,Dayhoff -mrate E,I,G,I+G -mfreq FU -wbtl) [90]. The resulting tree file was imported into iToL for visualization and collapsing of nodes followed by final modifications in Affinity Designer [91].

### Concatenated protein trees for dissimilatory sulfate reduction genes

To create the concatenated protein trees for *asrABC* and *dsrAB*, reference sequences for each set of genes from a diverse set of environments were utilized [47]. Hmmsearch (version 3.3.0) was used to identify asr and dsr sequences in the database of predicted ORFs [80]. The identified genes were extracted from the predicted ORFs and concatenation and tree building were done according to the protocol previously described for the ribosomal proteins.

### Summary calculations and statistical analysis for association of sulfur genes with disease state and stage of CRC

Summary calculations of the total number of MAGs with the gene of interest per disease state, mean, median, and standard deviations were performed in R. Summary information can be found in Supplemental Table 3. To identify potential associations between the presence of specific microbial sulfur genes and disease state, chi-squared tests were performed. First, for each gene, each participant was binarized as either “presence” (at least 1 MAG with at least 1 copy of the gene of interest) or “absence” (no MAGs recovered from the participant’s sample had any copies of the gene of interest). For each gene, total presence and absence were tallied for each disease state (healthy, adenoma, carcinoma). Chi-square tests were performed for each gene on a 2 × 3 matrix of presence/absence totals and disease states. *p*-value corrections were done using the Benjamini-Hochberg (BH) Procedure for final reported *p*-values. A significant *p*-value indicates the distribution of the gene among the disease states is not random. Summary values and statistics were all performed in R. Uncorrected and corrected p-values can be found in Supplemental Table 6. Dot plot visualizations with the proportion of participants in each of the three disease states with at least one copy of the gene in their MAGs normalized to the total number of participants with that disease state as the size of the dot and mean number of MAGs with a copy of the gene per participant with at least one copy as the color of the dot were made using R with the cowplot package. Data used for these plots can be found in Supplemental Table 3.

Similarly, to identify associations between the presence of specific microbial sulfur genes and colorectal cancer stage, the subset of studies with staging data was binarized as described above and binomial logistic regressions were performed. Rao score tests were performed on the resulting binomial logistic regressions to evaluate for the significance of each gene having evidence of association with staging of CRC. *p*-value corrections were done using the Benjamini-Hochberg (BH) Procedure for final reported *p*-values. Uncorrected and corrected p-values can be found in Supplemental Table 7. Presence/absence values for each gene in each stage were normalized to the total number of participants in that particular stage to generate distribution plots (Fig. S6).

### Metabolic reconstruction of MAGs

Metabolic reconstruction of each MAG was accomplished using the METABOLIC-G program of METABOLIC (version 4.0) [79]. Summary information is available at https://github.com/escowley/HumanGutBacterialSulfurCycle.

### Determination of growth rates for colorectal cancer indicator bacteria

To determine the growth rates of bacteria previously implicated as indicators of colorectal cancer in the gut community [11, 17], the original reads were downloaded and used to generate these genomes from the Hannigan, Yu, Zeller, and Feng studies. For the reads from the Hannigan study, the fasterq-dump (version 2.9.4) module of the SRA toolkit [92] was used, for the reads from the Yu and Zeller studies, the Aspera [93] command-line interface (CLI) ascp program (v3.9.1.168954) was used, and for the reads from the Feng study, the Aspera CLI ascp program (v3.9.3.177167) was used. For reads from the Zeller study, multiple read sets are deposited for each participant, and the first listed paired-end read set for each genome listed in the European Nucleotide Archive (ENA) metadata was chosen. Reads from the Hannigan study were trimmed and quality filtered with metaWRAP (v1.2.2) using the read_qc [94] module with the option “--skip-bmtagger”. Bowtie2 (v2.3.4.1) [95–97] was used with the option “--reorder” to map MAGs classified as those bacteria to the original read sets and shrinksam [98] version 0.9.0 with the “-u” flag to compress mapping files. Growth rates were determined by generating un-filtered indexes of replication with iRep (version 1.10) [99]. Growth rates for the following organisms were determined based on the closest taxonomic designator: *Fusobacterium mortiferum*, *Fusobacterium ulcerans*, *Fusobacterium nucleatum*, *Desulfovibrio piger*, *Bacteroides fragilis*, *Escherichia coli*, *Pyramidobacter piscolens*, *Clostridium difficile*, *Clostridium hylemonae*, *Porphyromonas asaccharolytica*, *Peptostreptococcus stomatis*, *Bilophila wadsworthia*, and *Odoribacter splanchnicus* (Table S8). Genomes from the species *F. nucleatum*, *P. piscolens*, and *O. splanchnicus* were not plotted. To determine differences in growth rates between CRC and healthy samples for all 3 studies, a Kruskal Wallis test was performed for each organism. To determine differences in growth rates across all disease states within a study for a particular organism, a Kruskal Wallis test was performed for each organism. *p*-value corrections were done using the Benjamini-Hochberg (BH) Procedure for final reported *p*-values. Uncorrected and corrected *p*-values can be found in Table S9. Code for statistical analysis and generation of plots can be found at https://github.com/escowley/HumanGutBacterialSulfurCycle.

## Supplementary Information

**Additional file 1: Supplemental Figure 1.** Concatenated ribosomal protein tree of all samples demonstrating the full diversity of samples included in our analyses. **Supplemental Figure 2.** Sulfidogenic functional genes are widespread in the human gut microbiome. **Supplemental Figure 3.** Comprehensive dot plots of (A) highly-abundant and (B) all other genes analyzed related to bacterial taurine and sulfur metabolism across three disease states: healthy, adenoma, and carcinoma. **Supplemental Figure 4.** Sulfidogenic pathways are significantly associated with colorectal cancer. **Supplemental Figure 5.** Cysteine-metabolizing genes are abundant in bacteria commonly associated with CRC. **Supplemental Figure 6.** Genes for bacterial sulfur metabolism are differentially distributed across different stages of colorectal cancer. **Supplemental Figure 7.** Indicator species for CRC are more likely to be present in participants with CRC and there are no differences in growth rates across disease states for any organism analyzed.

**Additional file 2: Supplemental Table 1.** Summary information for genes discussed in this study including abbreviations, full gene name, reaction the gene catalyzes, and HMM information. **Supplemental Table 2.** Summary table of HMM results where “gene” indicates that gene was found in the MAG and “N/A” indicates the gene was not found in the MAG. **Supplemental Table 3.** Summary values of participants and MAGs with and without sulfur genes. These data were used to generate dot plots. **Supplemental Table 4.** Summary of MAG genera and gene hits associated with each genera from the study in addition to any previous associations of that genera with colorectal cancer and production of H2S. **Supplemental Table 5.** MAGs associated with complete and partial taurine pathways. **Supplemental Table 6.** Results from chi squared tests for associations of gene presence with participant disease status. Both uncorrected and corrected *p*-values are reported. **Supplemental Table 7.** Results from ANOVA tests for associations between presence and absence of sulfur genes and stage of colorectal cancer. Both uncorrected and corrected p-values are reported. **Supplemental Table 8.** Growth rate values generated by iRep for indicator species of colorectal cancer for 4 of the 5 studies used in our work. **Supplemental Table 9.** Results from Kruskal Wallis analysis for differences in growth rates for the aggregate data for CRC versus healthy and each individual study including comparisons across CRC, healthy, and adenoma, if present. **Supplemental Table 10.** Summary of taxonomy of MAGs including classification from original paper constructing MAGs (assigned and closest designators), SILVA classification based on 16S rRNA sequences, and GTDB-tk classifications.

## Data Availability

Raw reads for the original metagenomic studies are deposited at https://www.ebi.ac.uk/ena/browser/view/PRJEB12449, https://www.ebi.ac.uk/ena/browser/view/PRJEB7774, https://www.ebi.ac.uk/ena/browser/view/PRJEB10878, https://www.ncbi.nlm.nih.gov/bioproject/PRJNA389927/, and https://www.ebi.ac.uk/ena/browser/view/PRJEB6070. MAGs were constructed previously [35] and were used for this study (see “downloading bins and accessing metadata” section of the methods). Code and HMM profiles are available at https://github.com/escowley/HumanGutBacterialSulfurCycle.

## References

[1] Tremaroli V, Bäckhed F (2012). Functional interactions between the gut microbiota and host metabolism. Nature.

[2] Clemente JC, Ursell LK, Parfrey LW, Knight R (2012). The impact of the gut microbiota on human health: an integrative view. Cell.

[3] Human Microbiome Project Consortium (2012). Structure, function and diversity of the healthy human microbiome. Nature.

[4] Raibaud O, Goldberg ME. The tryptophanase from *Escherichia coli* K-12. II. Comparison of the thermal stabilities of apo-, holo-, and hybrid enzymes. J Biol Chem. 1973;248:3451–5. 4573978

[5] Warren YA, Citron DM, Merriam CV, Goldstein EJC. Biochemical differentiation and comparison of *Desulfovibrio* species and other phenotypically similar genera. J Clin Microbiol. 2005;43:4041–5. 10.1128/JCM.43.8.4041-4045.2005 PMC1233901 16081948

[6] Lloyd-Price J, Mahurkar A, Rahnavard G, Crabtree J, Orvis J, Hall AB (2017). Strains, functions and dynamics in the expanded Human Microbiome Project. Nature.

[7] Islami F, Goding Sauer A, Miller KD, Siegel RL, Fedewa SA, Jacobs EJ (2018). Proportion and number of cancer cases and deaths attributable to potentially modifiable risk factors in the United States. CA Cancer J Clin.

[8] David LA, Maurice CF, Carmody RN, Gootenberg DB, Button JE, Wolfe BE (2014). Diet rapidly and reproducibly alters the human gut microbiome. Nature.

[9] O’Keefe SJD, Li JV, Lahti L, Ou J, Carbonero F, Mohammed K (2015). Fat, fibre and cancer risk in African Americans and rural Africans. Nat Commun.

[10] Devkota S, Wang Y, Musch MW, Leone V, Fehlner-Peach H, Nadimpalli A, et al. Dietary-fat-induced taurocholic acid promotes pathobiont expansion and colitis in Il10 ^−/−^ mice. Nature. 2012;487:104–8. 10.1038/nature11225 PMC3393783 22722865

[11] Yazici C, Wolf PG, Kim H, Cross T-WL, Vermillion K, Carroll T (2017). Race-dependent association of sulfidogenic bacteria with colorectal cancer. Gut.

[12] Kostic AD, Chun E, Robertson L, Glickman JN, Gallini CA, Michaud M, et al. *Fusobacterium nucleatum* potentiates intestinal tumorigenesis and modulates the tumor-immune microenvironment. Cell Host Microbe. 2013;14:207–15. 10.1016/j.chom.2013.07.007 PMC3772512 23954159

[13] Gao Z, Guo B, Gao R, Zhu Q, Qin H (2015). Microbiota dysbiosis is associated with colorectal cancer. Front Microbiol.

[14] Kostic AD, Gevers D, Pedamallu CS, Michaud M, Duke F, Earl AM, et al. Genomic analysis identifies association of *Fusobacterium* with colorectal carcinoma. Genome Res. 2012;22:292–8. 10.1101/gr.126573.111 PMC3266036 22009990

[15] Chen W, Liu F, Ling Z, Tong X, Xiang C (2012). Human intestinal lumen and mucosa-associated microbiota in patients with colorectal cancer. PLoS One.

[16] Castellarin M, Warren RL, Freeman JD, Dreolini L, Krzywinski M, Strauss J, et al. *Fusobacterium nucleatum* infection is prevalent in human colorectal carcinoma. Genome Res. 2012;22:299–306. 10.1101/gr.126516.111 PMC3266037 22009989

[17] Zeller G, Tap J, Voigt AY, Sunagawa S, Kultima JR, Costea PI (2014). Potential of fecal microbiota for early-stage detection of colorectal cancer. Mol Syst Biol.

[18] Wolf PG. Microbial pathways of sulfur metabolism and colorectal cancer risk [PhD Dissertation]: University of Illinois Urbana-Champaign; 2018.

[19] Huycke MM, Gaskins HR (2004). Commensal bacteria, redox stress, and colorectal cancer: mechanisms and models. Exp Biol Med.

[20] Furne J, Saeed A, Levitt MD (2008). Whole tissue hydrogen sulfide concentrations are orders of magnitude lower than presently accepted values. Am J Physiol Regul Integr Comp Physiol.

[21] Yang G, Wu L, Jiang B, Yang W, Qi J, Cao K, et al. H_2_S as a physiologic vasorelaxant: hypertension in mice with deletion of cystathionine -lyase. Science. 2008;322:587–90. 10.1126/science.1162667 PMC2749494 18948540

[22] Zheng J, Zhao T, Yuan Y, Hu N, Tang X. Hydrogen sulfide (H_2_S) attenuates uranium-induced acute nephrotoxicity through oxidative stress and inflammatory response via Nrf2-NF-κB pathways. Chem Biol Interact. 2015;242:353–62. 10.1016/j.cbi.2015.10.021 26523793

[23] Guo C, Liang F, Shah Masood W, Yan X (2014). Hydrogen sulfide protected gastric epithelial cell from ischemia/reperfusion injury by Keap1 s-sulfhydration, MAPK dependent anti-apoptosis and NF-κB dependent anti-inflammation pathway. Eur J Pharmacol.

[24] Attene-Ramos MS, Wagner ED, Plewa MJ, Gaskins HR (2006). Evidence that hydrogen sulfide is a genotoxic agent. Mol Cancer Res.

[25] Attene-Ramos MS, Wagner ED, Gaskins HR, Plewa MJ (2007). Hydrogen sulfide induces direct radical-associated DNA damage. Mol Cancer Res MCR.

[26] Attene-Ramos MS, Nava GM, Muellner MG, Wagner ED, Plewa MJ, Gaskins HR (2010). DNA damage and toxicogenomic analyses of hydrogen sulfide in human intestinal epithelial FHs 74 Int cells. Environ Mol Mutagen.

[27] Deplancke B, Gaskins HR (2003). Hydrogen sulfide induces serum-independent cell cycle entry in nontransformed rat intestinal epithelial cells. FASEB J.

[28] Carbonero F, Benefiel AC, Alizadeh-Ghamsari AH, Gaskins HR (2012). Microbial pathways in colonic sulfur metabolism and links with health and disease. Front Physiol.

[29] Ridlon JM, Wolf PG, Gaskins HR (2016). Taurocholic acid metabolism by gut microbes and colon cancer. Gut Microbes.

[30] Nguyen LH, Ma W, Wang DD, Cao Y, Mallick H, Gerbaba TK (2020). Association between sulfur-metabolizing bacterial communities in stool and risk of distal colorectal cancer in men. Gastroenterology.

[31] Dordević D, Jančíková S, Vítězová M, Kushkevych I (2021). Hydrogen sulfide toxicity in the gut environment: meta-analysis of sulfate-reducing and lactic acid bacteria in inflammatory processes. J Adv Res.

[32] Feng Q, Liang S, Jia H, Stadlmayr A, Tang L, Lan Z (2015). Gut microbiome development along the colorectal adenoma–carcinoma sequence. Nat Commun.

[33] Vogtmann E, Hua X, Zeller G, Sunagawa S, Voigt AY, Hercog R (2016). Colorectal cancer and the human gut microbiome: Reproducibility with whole-genome shotgun sequencing. PLoS One.

[34] Hannigan GD, Duhaime MB, Ruffin MT, Koumpouras CC, Schloss PD (2018). Diagnostic potential and interactive dynamics of the colorectal cancer virome. mBio.

[35] Yu J, Feng Q, Wong SH, Zhang D, yi Liang Q, Qin Y (2017). Metagenomic analysis of faecal microbiome as a tool towards targeted non-invasive biomarkers for colorectal cancer. Gut.

[36] Pasolli E, Asnicar F, Manara S, Zolfo M, Karcher N, Armanini F (2019). Extensive unexplored human microbiome diversity revealed by over 150,000 genomes from metagenomes spanning age, geography, and lifestyle. Cell.

[37] Rabus R, Hansen TA, Widdel F. Dissimilatory sulfate- and sulfur-reducing prokaryotes. In: Rosenberg E., DeLong E.F., Lory S., Stackebrandt E., Thompson F. (eds) The Prokaryotes. Heidelberg: Springer. 2013. 10.1007/978-3-642-30141-4_70.

[38] Florin T, Neale G, Gibson GR, Christl SU, Cummings JH (1991). Metabolism of dietary sulphate: absorption and excretion in humans. Gut.

[39] Alnouti Y (2009). Bile acid sulfation: a pathway of bile acid elimination and detoxification. Toxicol Sci.

[40] Corfield AP, Wagner SA, Clamp JR, Kriaris MS, Hoskins LC (1992). Mucin degradation in the human colon: production of sialidase, sialate O-acetylesterase, N-acetylneuraminate lyase, arylesterase, and glycosulfatase activities by strains of fecal bacteria. Infect Immun.

[41] Lengeler JW, Drews G, Schlegel HG. Biology of the Prokaryotes: Wiley-Blackwell; 1999.

[42] Müller AL, Kjeldsen KU, Rattei T, Pester M, Loy A (2015). Phylogenetic and environmental diversity of DsrAB-type dissimilatory (bi)sulfite reductases. ISME J.

[43] Gibson GR, Macfarlane GT, Cummings JH (1988). Occurrence of sulphate-reducing bacteria in human faeces and the relationship of dissimilatory sulphate reduction to methanogenesis in the large gut. J Appl Bacteriol.

[44] Nava GM, Carbonero F, Croix JA, Greenberg E, Gaskins HR (2012). Abundance and diversity of mucosa-associated hydrogenotrophic microbes in the healthy human colon. ISME J.

[45] Zinkevich V, Beech IB (2000). Screening of sulfate-reducing bacteria in colonoscopy samples from healthy and colitic human gut mucosa. FEMS Microbiol Ecol.

[46] Fite A, Macfarlane GT, Cummings JH, Hopkins MJ, Kong SC, Furrie E (2004). Identification and quantitation of mucosal and faecal desulfovibrios using real time polymerase chain reaction. Gut.

[47] Anantharaman K, Hausmann B, Jungbluth SP, Kantor RS, Lavy A, Warren LA (2018). Expanded diversity of microbial groups that shape the dissimilatory sulfur cycle. ISME J.

[48] Lie TJ, Pitta T, Leadbetter ER, Godchaux W, Leadbetter JR (1996). Sulfonates: novel electron acceptors in anaerobic respiration. Arch Microbiol.

[49] Lie TJ, Godchaux W, Leadbetter ER. Sulfonates as terminal electron acceptors for growth of sulfite-reducing bacteria (*Desulfitobacterium* spp.) and sulfate-reducing bacteria: effects of inhibitors of sulfidogenesis. Appl Environ Microbiol. 1999;65:4611–7. 10.1128/aem.65.10.4611-4617.1999 PMC91615 10508097

[50] Laue H, Denger K, Cook AM. Taurine reduction in anaerobic respiration of *Bilophila wadsworthia* RZATAU. Appl Environ Microbiol. 1997;63:2016–21. 10.1128/aem.63.5.2016-2021.1997 PMC168491 9143131

[51] Ahlman B, Leijonmarck C-E, Wernerman J (1993). The content of free amino acids in the human duodenal mucosa. Clin Nutr.

[52] Islam KBMS, Fukiya S, Hagio M, Fujii N, Ishizuka S, Ooka T (2011). Bile acid is a host factor that regulates the composition of the cecal microbiota in rats. Gastroenterology.

[53] Albenberg L, Esipova TV, Judge CP, Bittinger K, Chen J, Laughlin A (2014). Correlation between intraluminal oxygen gradient and radial partitioning of intestinal microbiota. Gastroenterology.

[54] Peck SC, Denger K, Burrichter A, Irwin SM, Balskus EP, Schleheck D. A glycyl radical enzyme enables hydrogen sulfide production by the human intestinal bacterium *Bilophila wadsworthia*. Proc Natl Acad Sci. 2019;116:3171–6. 10.1073/pnas.1815661116 PMC6386719 30718429

[55] Wu G, Shi Y, Han L, Feng C, Ge Y, Yu Y (2020). Dietary methionine restriction ameliorated fat accumulation, systemic inflammation, and increased energy metabolism by altering gut microbiota in middle-aged mice administered different fat diets. J Agric Food Chem.

[56] Liu G, Yu L, Fang J, Hu C-AA, Yin J, Ni H (2017). Methionine restriction on oxidative stress and immune response in dss-induced colitis mice. Oncotarget.

[57] Shatalin K, Shatalina E, Mironov A, Nudler E. H_2_S: a universal defense against antibiotics in bacteria. Science. 2011;334:986–90. 10.1126/science.1209855 22096201

[58] Rizzo AA (1967). The possible role of hydrogen sulfide in human periodontal disease. I. Hydrogen sulfide production in periodontal pockets. Periodontics.

[59] Horowitz A, Folke LE (1973). Hydrogen sulfide production in the periodontal environment. J Periodontol.

[60] Ng W, Tonzetich J (1984). Effect of hydrogen sulfide and methyl mercaptan on the permeability of oral mucosa. J Dent Res.

[61] Yaegaki K, Qian W, Murata T, Imai T, Sato T, Tanaka T (2008). Oral malodorous compound causes apoptosis and genomic DNA damage in human gingival fibroblasts. J Periodontal Res.

[62] Marchesi JR, Dutilh BE, Hall N, Peters WHM, Roelofs R, Boleij A (2011). Towards the human colorectal cancer microbiome. PLoS One.

[63] McCoy AN, Araújo-Pérez F, Azcárate-Peril A, Yeh JJ, Sandler RS, Keku TO. *Fusobacterium* is associated with colorectal adenomas. PLoS One. 2013;8:e53653. 10.1371/journal.pone.0053653 PMC3546075 23335968

[64] Mima K, Sukawa Y, Nishihara R, Qian ZR, Yamauchi M, Inamura K, et al. *Fusobacterium nucleatum* and T cells in colorectal carcinoma. JAMA Oncol. 2015;1:653–61. 10.1001/jamaoncol.2015.1377 PMC4537376 26181352

[65] Mima K, Nishihara R, Qian ZR, Cao Y, Sukawa Y, Nowak JA, et al. *Fusobacterium nucleatum* in colorectal carcinoma tissue and patient prognosis. Gut. 2016;65:1973–80. 10.1136/gutjnl-2015-310101 PMC4769120 26311717

[66] Florin THJ (1991). Hydrogen sulphide and total acid-volatile sulphide in faeces, determined with a direct spectrophotometric method. Clin Chim Acta.

[67] Chacko A, Cummings JH (1988). Nitrogen losses from the human small bowel: obligatory losses and the effect of physical form of food. Gut.

[68] Scanlan PD, Shanahan F, Marchesi JR (2009). Culture-independent analysis of desulfovibrios in the human distal colon of healthy, colorectal cancer and polypectomized individuals. FEMS Microbiol Ecol.

[69] Cai W-J, Wang M-J, Ju L-H, Wang C, Zhu Y-C (2010). Hydrogen sulfide induces human colon cancer cell proliferation: role of Akt, ERK and p21. Cell Biol Int.

[70] Winter SE, Thiennimitr P, Winter MG, Butler BP, Huseby DL, Crawford RW, et al. Gut inflammation provides a respiratory electron acceptor for *Salmonella*. Nature. 2010;467:426–9. 10.1038/nature09415 PMC2946174 20864996

[71] Braccia DJ, Jiang X, Pop M, Hall AB. The capacity to produce hydrogen sulfide (H_2_S) via cysteine degradation is ubiquitous in the human gut microbiome. Frontiers in Microbiology. 2021;12. https://www.frontiersin.org/articles/10.3389/fmicb.2021.705583/full. 10.3389/fmicb.2021.705583 PMC8564485 34745023

[72] Markowitz VM, Chen I-MA, Chu K, Szeto E, Palaniappan K, Jacob B (2012). IMG/M-HMP: a metagenome comparative analysis system for the human microbiome project. PLoS One.

[73] Turnbaugh PJ, Ley RE, Hamady M, Fraser-Liggett CM, Knight R, Gordon JI (2007). The human microbiome project. Nature.

[74] Altschul SF, Gish W, Miller W, Myers EW, Lipman DJ (1990). Basic local alignment search tool. J Mol Biol.

[75] Thomas AM, Manghi P, Asnicar F, Pasolli E, Armanini F, Zolfo M (2019). Metagenomic analysis of colorectal cancer datasets identifies cross-cohort microbial diagnostic signatures and a link with choline degradation. Nat Med.

[76] Wirbel J, Pyl PT, Kartal E, Zych K, Kashani A, Milanese A (2019). Meta-analysis of fecal metagenomes reveals global microbial signatures that are specific for colorectal cancer. Nat Med.

[77] Bowers RM, Kyrpides NC, Stepanauskas R, Harmon-Smith M, Doud D, Reddy TBK (2017). Minimum information about a single amplified genome (MISAG) and a metagenome-assembled genome (MIMAG) of bacteria and archaea. Nat Biotechnol.

[78] Hyatt D, Chen G-L, LoCascio PF, Land ML, Larimer FW, Hauser LJ (2010). Prodigal: prokaryotic gene recognition and translation initiation site identification. BMC Bioinformatics.

[79] Zhou Z, Tran PQ, Breister AM, Liu Y, Kieft K, Cowley ES, et al. METABOLIC: high-throughput profiling of microbial genomes for functional traits, metabolism, biogeochemistry, and community-scale functional networks. Microbiome. 2022;10:33. https://microbiomejournal.biomedcentral.com/articles/10.1186/s40168-021-01213-8. 10.1186/s40168-021-01213-8 PMC8851854 35172890

[80] HMMER. Available from: http://hmmer.org/. Cited 2021 Apr 1.

[81] Parks DH, Imelfort M, Skennerton CT, Hugenholtz P, Tyson GW (2015). CheckM: assessing the quality of microbial genomes recovered from isolates, single cells, and metagenomes. Genome Res.

[82] Quast C, Pruesse E, Yilmaz P, Gerken J, Schweer T, Yarza P (2013). The SILVA ribosomal RNA gene database project: improved data processing and web-based tools. Nucleic Acids Res.

[83] Yilmaz P, Parfrey LW, Yarza P, Gerken J, Pruesse E, Quast C (2014). The SILVA and “All-species Living Tree Project (LTP)” taxonomic frameworks. Nucleic Acids Res.

[84] Pruesse E, Peplies J, Glöckner FO (2012). SINA: accurate high-throughput multiple sequence alignment of ribosomal RNA genes. Bioinformatics.

[85] Parks DH, Chuvochina M, Waite DW, Rinke C, Skarshewski A, Chaumeil P-A (2018). A standardized bacterial taxonomy based on genome phylogeny substantially revises the tree of life. Nat Biotechnol.

[86] Parks DH, Chuvochina M, Chaumeil P-A, Rinke C, Mussig AJ, Hugenholtz P (2020). A complete domain-to-species taxonomy for Bacteria and Archaea. Nat Biotechnol.

[87] Chaumeil P-A, Mussig AJ, Hugenholtz P, Parks DH (2020). GTDB-Tk: a toolkit to classify genomes with the Genome Taxonomy Database. Bioinformatics.

[88] Anantharaman K, Brown CT, Hug LA, Sharon I, Castelle CJ, Probst AJ (2016). Thousands of microbial genomes shed light on interconnected biogeochemical processes in an aquifer system. Nat Commun.

[89] Katoh K, Standley DM (2013). MAFFT multiple sequence alignment software version 7: improvements in performance and usability. Mol Biol Evol.

[90] Nguyen L-T, Schmidt HA, von Haeseler A, Minh BQ (2015). IQ-TREE: a fast and effective stochastic algorithm for estimating maximum-likelihood phylogenies. Mol Biol Evol.

[91] Letunic I, Bork P (2019). Interactive Tree Of Life (iTOL) v4: recent updates and new developments. Nucleic Acids Res.

[92] SRA-Tools - NCBI. Available from: http://ncbi.github.io/sra-tools/. Cited 2021 Jun 9.

[93] Aspera - Connect. 2019. Available from: https://www.ibm.com/aspera/connect. Cited 2021 Jun 9.

[94] MetaWRAP—a flexible pipeline for genome-resolved metagenomic data analysis | Microbiome | Full Text. Available from: https://microbiomejournal.biomedcentral.com/articles/10.1186/s40168-018-0541-1. Cited 2021 Jun 9. 10.1186/s40168-018-0541-1 PMC6138922 30219103

[95] Langmead B, Salzberg SL (2012). Fast gapped-read alignment with Bowtie 2. Nat Methods.

[96] Langmead B, Wilks C, Antonescu V, Charles R (2019). Scaling read aligners to hundreds of threads on general-purpose processors. Bioinformatics.

[97] Langmead B, Trapnell C, Pop M, Salzberg SL (2009). Ultrafast and memory-efficient alignment of short DNA sequences to the human genome. Genome Biol.

[98] Thomas B (2013). bcthomas/shrinksam.

[99] Brown CT, Olm MR, Thomas BC, Banfield JF (2016). Measurement of bacterial replication rates in microbial communities. Nat Biotechnol.

